# [^18^F]Nifene PET/CT Imaging in Mice: Improved Methods and Preliminary Studies of α4β2* Nicotinic Acetylcholinergic Receptors in Transgenic A53T Mouse Model of α-Synucleinopathy and Post-Mortem Human Parkinson’s Disease

**DOI:** 10.3390/molecules26237360

**Published:** 2021-12-04

**Authors:** Anthony-David T. Campoy, Christopher Liang, Reisha M. Ladwa, Krystal K. Patel, Ishani H. Patel, Jogeshwar Mukherjee

**Affiliations:** Preclinical Imaging, Department of Radiological Sciences, University of California-Irvine, Irvine, CA 92697, USA; atcampoy@uci.edu (A.-D.T.C.); liangc@uci.edu (C.L.); rladwa@uci.edu (R.M.L.); kkpatel11@gmail.com (K.K.P.); ishanipa@usc.edu (I.H.P.)

**Keywords:** [^18^F]nifene, Hualpha-Syn(A53T), transgenic mice, Parkinson’s disease, α-synucleinopathy, Lewy bodies, PET/CT imaging

## Abstract

We report [^18^F]nifene binding to α4β2* nicotinic acetylcholinergic receptors (nAChRs) in Parkinson’s disease (PD). The study used transgenic Hualpha-Syn(A53T) PD mouse model of α-synucleinopathy for PET/CT studies in vivo and autoradiography in vitro. Additionally, postmortem human PD brain sections comprising of anterior cingulate were used in vitro to assess translation to human studies. Because the small size of mice brain poses challenges for PET imaging, improved methods for radiosynthesis of [^18^F]nifene and simplified PET/CT procedures in mice were developed by comparing intravenous (IV) and intraperitoneal (IP) administered [^18^F]nifene. An optimal PET/CT imaging time of 30–60 min post injection of [^18^F]nifene was established to provide thalamus to cerebellum ratio of 2.5 (with IV) and 2 (with IP). Transgenic Hualpha-Syn(A53T) mice brain slices exhibited 20–35% decrease while in vivo a 20–30% decrease of [^18^F]nifene was observed. Lewy bodies and α-synuclein aggregates were confirmed in human PD brain sections which lowered the [^18^F]nifene binding by more than 50% in anterior cingulate. Thus [^18^F]nifene offers a valuable tool for PET imaging studies of PD.

## 1. Introduction

**Figure 1:**
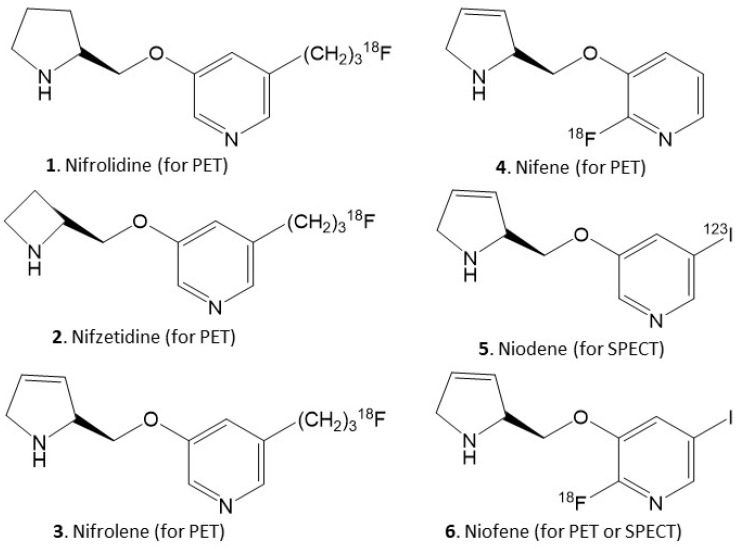
Chemical structures of PET and SPECT radiotracers for α4β2* nAChRs. **1**. [^18^F]Nifrolidine (PET); **2**. [^18^F]nifzetidine (PET); **3**. [^18^F]nifrolene (PET); **4**. [^18^F]nifene (PET); **5**. [^123^I]niodene (SPECT); **6**. [^18^F] or [^123^I]niofene (PET or SPECT).

Dysfunction of neuronal α4β2* nicotinic cholinergic receptors (nAChRs) has been implicated in numerous pathologies, including Parkinson’s disease (PD). Due to this clinical importance of α4β2* nAChRs, evaluating these receptors can potentially assist in the treatment of these human diseases. In order to image these receptors using positron emission tomography (PET) and single photon emission computed tomography (SPECT), we developed several α4β2* nAChRs fluorine-18 and iodine-123 labeled radiotracers (Figure 1). These include 5-fluoropropyl derivatives, [^18^F]nifrolidine [1], [^18^F]nifzetidine [2], [^18^F]nifrolene [3]. The fluoroalkyl group at the 5-position renders antagonist properties at the α4β2* nAChRs. The difference in the three radiotracers was the secondary amine ring, which dictated the in vivo binding kinetics. [^18^F]Nifrolene was found most suitable with PET scan times of 90 min to equilibrate and good target to nontarget ratios in brain regions. [^18^F]Nifene 4 which lacks the fluoropropyl group, is more nicotine-like and has more agonist like properties [4,5]. Imaging of α4β2* nAChRs by SPECT is made possible by inclusion of iodine-123 at the 5-position in [^123^I]niodene [6]. Both PET and SPECT studies with the identical molecule was made possible by using [^18^F]niofene or [^123^I]niofene, which contains both a fluorine and iodine substituent [7].

Of the structures shown in Figure 1, we have taken [^18^F]nifene to human PET studies. Nifene binds selectively to the β2 subtype nAChRs, with binding affinities, Ki = 0.83 nM, 0.80 nM and 0.34 nM for α4β2, α3β2 and α2β2 nAChRs, respectively [5]. [^18^F]Nifene yielded the fastest in vivo equilibration in human PET studies amongst the various radiotracers currently being used for imaging α4β2* nAChRs [8]. A shorter PET scan time is important for patient comfort and improve subject compliance, minimize motion artifacts which can be detrimental in higher resolution scanners, and use less PET scanner time. [^18^F]Nifene has been shown to be superior in terms of imaging times in all species, providing reasonable target-to-nontarget ratios and providing the ability to visualize extrathalamic receptors [5]. Biological properties of [^18^F]nifene reported in rodents [9,10], monkeys [4,11] and humans [8] suggest some differences in binding of [^18^F]nifene across the different species. Additional constraints in the different species occur due to resolution of scanners for a mouse (~25 g), rat (~250 g), monkey (~10,000 g) and human (~70,000 g) as previously discussed for [^18^F]nifene [5].

Cholinergic impairments may occur in PD, a neurodegenerative disease with motor and non-motor symptoms [12,13]. The sporadic nature of PD development can make studying specific pathologies challenging [14]. The endogenous protein α-synuclein, is generally structured as a random coil but in PD, the misfolded α-synuclein aggregates in neurons [15]. Aggregation of misfolded α-synuclein in intracellular inclusions and Lewy bodies (LB) are some of the hallmarks of human PD [16]. Glucose metabolism studies using [^18^F]FDG-PET have been successfully carried out in human PD [17,18]. With [^18^F]FDG-PET, specific patterns of deficit in the PD brain can be identified [19,20,21].

Several transgenic mice models of α-synucleinopathy allow the study of PD progression [22]. Using PET/CT studies in Hualpha-Syn(A53T) α-synuclein mutant mice, we recently reported [^18^F]FDG brain deficits, limb muscle deficits and reduction of metabolic activity in regions of the spinal cord [23]. These findings are consistent with the increased α-synuclein expression in the Hualpha-Syn(A53T) transgenic mice and spontaneously develop neurodegenerative disease between nine to 16 months of age [24,25]. Similar to human PD, neuronal abnormalities include pathological accumulations of α-synuclein and ubiquitin and Lewy body inclusions in neurons, with profound deficits in their motor neurons resulting in paralysis [22]. Our preliminary findings suggested hypometabolism in the lower spinal cord suggesting degeneration as a probable cause for the paralysis [23]. The Hualpha-Syn(A53T) mice also develop fine, sensorimotor, and synaptic deficits before developing age-related gross motor and cognitive impairment [25]. Higher levels of acetylcholine have been reported in α-synuclein transgenic mice [26].

Because of the cholinergic role in PD, we have now investigated binding of [^18^F]nifene in Hualpha-Syn(A53T) α-synuclein mutant mice and postmortem human PD. Both in vitro and in vivo studies were caried out in Hualpha-Syn(A53T). Preliminary [^18^F]nifene in postmortem human PD brains comprising of anterior cingulate were carried out to assess potential alterations in α4β2*nAChRs. In order to establish the use of [^18^F]nifene imaging in mice, we report here the following: (1) Improved radiosynthesis using a new trimethylammonium salt precursor; (2) PET/CT studies in normal mice and evaluation of quantitation methods using mouse brain MRI template; (3) evaluation of [^18^F]nifene binding in α-synucleinopathy mice model of PD; and (4) binding in post-mortem human anterior cingulate brain sections of control and PD subjects.

## 2. Materials and Methods

### 2.1. General Methods

All chemicals and solvents were purchased from Aldrich Chemical and Fisher Scientific. Deionized water was acquired from Millipore Milli-Q Water Purification System (Burlington, MA, USA). Fluorine-18 fluoride in oxygen-18 enriched water was purchased from PETNET Inc. (Riverside, CA, USA), Fluorine-18 radioactivity was counted in a Capintec CRC-15R dose calibrator (Florham Park, NJ, USA) while low level counting was carried out in a Capintec Caprac-R well-counter (Florham Park, NJ, USA). All solvents used were provided by Fisher Scientific (Waltham, MA, USA). Gilson high performance liquid chromatography (HPLC) was used for the semi-preparative reverse-phase column chromatography with UV detector set at dual wavelengths of 254 and 280 nm as well as a radioactivity detector (Middleton, WI, USA). A Semi-preparative HPLC column 100 × 250 mm 10 micron Econosil C18 reverse-phase was used. Analytical thin-layer chromatography (TLC) was used to monitor reactions (Baker-flex, Phillipsburg, NJ, USA). RadioTLC were scanned on an AR-2000 imaging scanner (Eckart and Ziegler, Berlin, Germany). Electrospray mass spectra were obtained from a Model 7250 mass spectrometer (Micromass LCT, Waters Corp., Milford, MA, USA). Proton NMR spectra were recorded on a Bruker OM EGA 500-MHz spectrometer (Billerica, MA, USA). Mice brain slices were prepared at 10 to 40 µm thick using the Lieca 1850 cryotome. In vitro- or ex vivo-labeled brain sections were exposed to phosphor films (Perkin Elmer Multisensitive, Medium MS) and read using the Cyclone Phosphor Imaging System (Waltham, MA, USA). Analysis of autoradiographs was done using Optiquant acquisition and analysis software (Perkin Elmer, Waltham, MA, USA).

### 2.2. Animals

All animal studies were approved by the Institutional Animal Health Care and Use Committee of University of California-Irvine.

#### 2.2.1. BALB/c Mice

Female mice (n = 4) age 14–18 weeks were used for time–activity of [^18^F]nifene in this study (24 g). Mice were purchased from Jackson Laboratory and housed under controlled temperatures of 22 ± 1 °C, in a 12 h light–dark cycle, on at 6:00 a.m., with water and food chow ad libitum.

#### 2.2.2. C57BL/6 Mice

Adult male mice (n = 4) were used in this study (28 g; 18–24 weeks). Mice were purchased from Jackson Laboratory and housed under controlled temperatures of 22 ± 1 °C, in a 12 h light–dark cycle, on at 6:00 a.m., with water and food chow ad libitum.

#### 2.2.3. Hualpha-Syn (A53T) Transgenic Mice

The Hualpha-Syn (A53T) transgenic line of mice (Tg(Prnp-SNCA*A53T) 83 Vle/J; stock no. 004479; 4 male and 4 female) and non-carrier mice (4 male and 4 female) were purchased from Jackson Laboratory. Female mice were 20–28 g and male mice weighed 26–38 g and were 11 months old. All mice were housed in sterilized cages. Non-carrier animals did not exhibit any abnormal motor activity whereas Hualpha-Syn (A53T) mice over time had hind limb paralysis and were euthanized when necessary. All animals recovered from the anesthesia required for the PET/CT imaging procedures.

### 2.3. Human Tissue

Human postmortem brain tissue samples were obtained from Banner Sun Health Research Institute (BHRI), Sun City, AZ brain tissue repository for in vitro experiments. Age and gender matched PD brain and cognitively normal (CN) brain tissue samples were used for the study. Human postmortem brain slices were obtained from chunks of frozen tissue on a Leica 1850 cryotome cooled to −20 °C. Fluorine-18 autoradiographic studies were carried out by exposing tissue samples on storage phosphor screens (Perkin Elmer Multisensitive, Medium MS and tritium sensitive phosphor screens). The apposed phosphor screens were read and analyzed by OptiQuant acquisition and analysis program of the Cyclone Storage Phosphor System (Packard Instruments Co., Boston, MA, USA). Adjacent slices were used for immunostaining with anti-ubiquitin and anti-α-synuclein. All postmortem human brain studies were approved by the Institutional Biosafety Committee of University of California, Irvine.

### 2.4. Synthesis

2-(Trimethylamino)-3-[2-((*S*)-*N*-tert-butoxycarbonyl-3-pyrrolinyl)methoxy]pyridine Triflate (TMAT) 8: Synthesis of *N*-BOC-nifene was carried out using our procedures reported previously in >95% purity, [4]). *N*-BOC-nifene (0.3 g) was dissolved in 10 mL of dimethylamine in a Wheaton Kimble V-vial. This vial was heated at 100 °C for 23 h in a Lab-Line Multi-Blok heater. After it was cooled to ambient temperature, the solvent was removed in vacuo. The oily residue was then extracted with dichloromethane and purified using preparative TLC in 9:1 dichloromethane-methanol solvent resulting in 2-(dimethylamino)-3-[2-((*S*)-*N*-tert-butoxycarbonyl-3-pyrrolinyl)methoxy]pyridine (0.2 g). MS, *m*/*z*, 320 (40%, [M + H]^+^), 342 (58%, [M + Na]^+^), 661 (100%, [2M + Na]^+^). Product 1: 1H NMR (500 MHz, CD_3_OD) δ ppm: 7.73 (dd, 1H, *J* = 5.04 and 4.98 Hz), 7.21 (d, 1H, *J* = 4.98 Hz), 6.80 (m, 1H), 5.93(s, 1H, olefinic), 5.94 (m, 1H), 4.25 (m, 2H), 3.32 (m, 2H), 2.93 (d, 6H, N(CH_3_)_2_) 1.46 (d, 9H, *J* = 5.64 Hz, BOC).

To a solution of 160 mg of 2-(dimethylamino)-3-[2-((*S*)-*N*-tert-butoxycarbonyl-3-pyrrolinyl)methoxy]pyridine in 2 mL of toluene, methyl trifluoromethanesulfonate was added (0.08 mL). The solution was stirred at room temperature for 1 h and then diluted with 10 mL of water and dichloromethane (0.5:9.5). The dichloromethane was extracted, dried over magnesium sulfate and filtered. The filtrate was concentrated to an oily residue and purified using preparative TLC in 9:1 dichloromethane-methanol solvent. The purified oily product, 2-(trimethylamino)-3-[2-((*S*)-*N*-tert-butoxycarbonyl-3-pyrrolinyl)methoxy]pyridine triflate (TMAT), 18.2 mg was obtained. MS, *m*/*z*, 278 (15%, [M − C_4_H_8_]^+^), 275 (23%, [M − NMe_3_]^+^), 234 (100%, [M − C_4_H_8_CO_2_]^+^). 1H NMR (500 MHz, CD_3_OD) δ ppm: 8.20 (d, 1H, *J* = 4.43 Hz), 7.94 (d, 1H, *J* = 8.27 Hz), 7.72 (dd, 1H, *J* = 4.51 and 4.54 Hz), 6.18 (m, 1H, olefinic), 6.02 (m, 1H, olefinic), 4.62 (d, 1H, *J* = 10.37), 4.43 (1H, d, *J* = 10.29 Hz), 4.13 (s, 1H), 3.52 (m, 2H), 2.26 (s, 9H, N(CH_3_)_3_) 1.29 (9H, s, BOC).

### 2.5. Radiosynthesis

The radiosynthesis of [^18^F]nifene was performed using nucleophilic displacement of the nitro group in *N*-BOC-nitronifene precursor by [^18^F]fluoride in an automated synthesizer followed by deprotection using previously described procedures for the various species [4,11]. The automated radiosynthesis of [^18^F]nifene was carried out in the chemistry-processing control unit (CPCU) box or the GE TRACERlab F X 2 N. High specific activity [^18^F]fluoride in H_2_^18^O from PETNET was passed through QMA-light Sep-Pak (Waters Corp, Milford, MA, USA), which was previously preconditioned with 2 mL of K_2_CO_3_ (140 mg/mL), followed by 2 mL of anhydrous acetonitrile. The trapped [^18^F]fluoride in QMA was eluted with 2.5 mL of Kryptofix-K_2_CO_3_ solution (36 mg/7.5 mg in 0.1 mL water and 2.4 mL of acetonitrile) and transferred to the reaction vessel. Initial step in the radiosynthesis involved drying the [^18^F]fluoride solution at 125 °C for 10 min. Subsequently, 2 × 1 mL of anhydrous acetonitrile was added to the reaction vessel for azeotropic removal of last traces of moisture by heating at 125 °C for 5 min each time.

**Figure 2:**
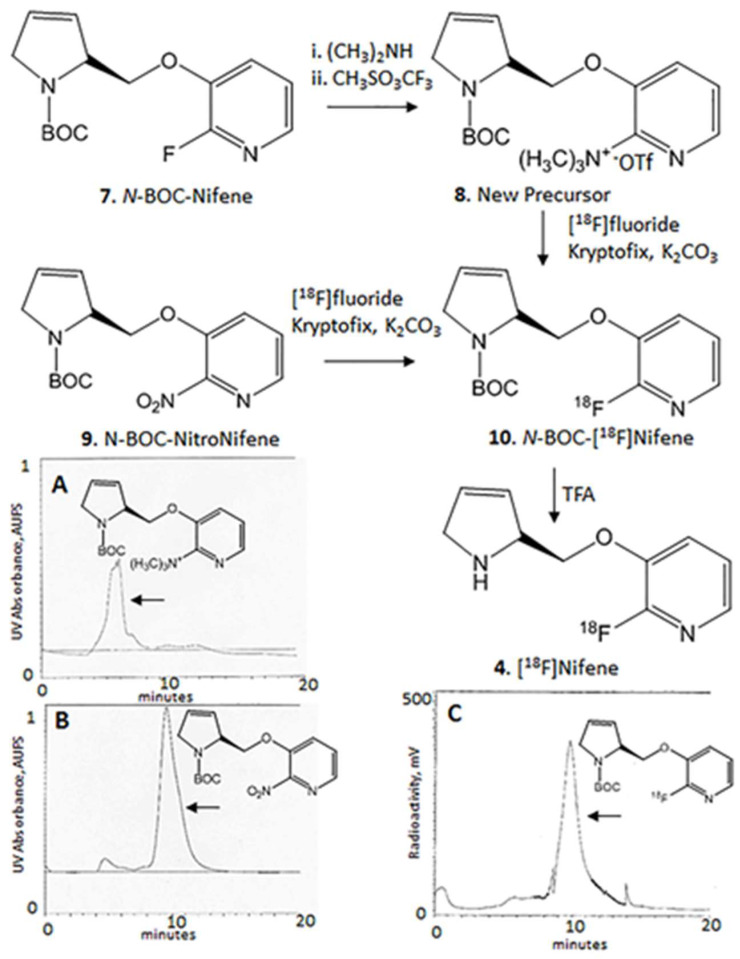
Improved [^18^F]nifene (4) synthesis. *N*-BOC-nifene (7) was converted to the 2-(trimethylamino)-3-[2-((*S*)-*N*-tert-butoxycarbonyl-3-pyrrolinyl)methoxy]pyridine triflate (TMAT) (8) by reacting with dimethylamine and methyl trifluorosulfonate. (**A**). HPLC chromatogram showing TMAT precursor retention time of approx. 6 min; (**B**). HPLC chromatogram showing *N*-BOC-nitronifene (9) precursor with retention time of approx. 9.5 min; (**C**). *N*-BOC-[^18^F]nifene (10) eluting at approx. 10 min on HPLC which is well separated from TMAT precursor, but close the *N*-BOC-nitronifene precursor.

The TMAT precursor 2-(trimethylamino)-3-[2-((*S*)-*N*-tert-butoxycarbonyl-3-pyrrolinyl)methoxy]pyridine triflate, 8 (2 mg dissolved in 0.2 mL anhydrous dimethylsulfoxide and 0.3 mL of anhydrous acetonitrile) or the nitro precursor, 2-nitro-3-[2-((*S*)-*N*-*tert*-butoxycarbonyl-3-pyrroline)methoxy]pyridine 9 (2 mg dissolved in 0.2 mL anhydrous dimethylsulfoxide and 0.3 mL of anhydrous acetonitrile) was transferred to the reaction vessel and the mixture was heated at 126 °C for 30 min for the nitro precursor and 15 min for the TMAT precursor. After the reaction, the contents of the reaction vessel were extracted by the addition of methanol (5 mL). This methanol extract was passed through neutral alumina to remove any unreacted [^18^F]fluoride in order to provide the intermediate *N*-BOC-[^18^F]nifene 10 (Figure 2). Reverse-phase HPLC purification of the methanol extract using an Alltech C_18_ column (10 μm, 250 × 10 mm), mobile phase: 60% acetonitrile-40% with 0.1% triethylamine, flow rate 2.5 mL/min, provided the product *N*-BOC-[^18^F]nifene 10 (Figure 2C). The collected fraction was taken to dryness in vacuo and the residue was taken in dichloromethane (1 mL) and TFA (0.2 mL). The mixture was heated at 80 °C (external temperature of heating block) for 30 min and subsequently evaporated to dryness. The residue was neutralized with 10% NaHCO_3_ to pH 7.0. The final formulation was carried out using 1-3 mL of saline (0.9% NaCl INJ) followed by sterile filtration through a membrane filter (0.22 μm), and filtered solution was collected in a dose vial. The radiolabeled product [^18^F]nifene (370–740 MBq) was obtained in >99% purity. Radiochemical yields of [^18^F]nifene with both the precursors were in the 40–50% range decay corrected, and specific activities ranged from 37–185 GBq/μmol and above.

### 2.6. BALB\c Mice PET and CT Scanning

Subjects had free access to food and water during housing. All animals were fasted for 18–24 h prior to PET imaging. In preparation for the scans, the mice were induced into anesthesia with 4% isoflurane. Inveon preclinical Dedicated PET (Siemen’s Inc., Erlangen, Germany) was used for the MicroPET studies which has a resolution of 1.45 mm [27]. The Inveon PET and MM CT scanners were placed in the “docked mode” for combined PET/CT experiments (Siemens Medical Solutions, Knoxville, TN, USA). A Sigma Delta anesthetic vaporizer (DRE, Louisville, KY, USA) was used to induce and maintain anesthesia during injections and PET/CT acquisitions.

Mice received intravenous (IV) using tail vein or intraperitoneal (IP) injections (approx. 7 MBq) of [^18^F]nifene off the scanner bed and then were immediately placed in the mouse imaging chamber for PET acquisition and scanned for 120 min in an Inveon dedicated PET scanner. The average delay between the time of injection and the start of the scan was 4 min. The animals were maintained under 2% isoflurane anesthesia throughout the scanning period. CT images were reconstructed with a cone beam algorithm (bilinear interpolation, Shepp-Logan filter) into 480 × 480 × 632 image arrays with a 206 μm pixel size. Following the reconstruction the CT images were spatially transformed to match the PET images. In addition to being reconstructed into an image, the CT data were used for attenuation correction of PET images. Quantitative calibration of PET images was performed by scanning 6.2 MBq of well mixed [^18^F]nifene and Millipore water solution inside a 56.5 mL plastic container. The [^18^F]nifene activity used in calibration was measured in the same dose calibrator used to measure the activities administered to the subjects.

### 2.7. C57BL\6 Mice PET and CT Scanning

Mice received intravenous (IV) using tail vein or intraperitoneal (IP) injections (approx. 7 MBq) of [^18^F]nifene off the scanner bed and then were immediately placed in the mouse imaging chamber for PET acquisition and scanned for 120 min in an Inveon dedicated PET scanner. The average delay between the time of injection and the start of the scan was 4 min. The animals were maintained under 2% isoflurane anesthesia throughout the scanning period.

#### 2.7.1. C57BL\6 Mice Ex Vivo Autoradiography

The brain after the ex vivo MicroPET acquisition in Section 2.5 was removed from the dry ice and rapidly prepared for sectioning. Horizontal sections (20 µm thick) containing [^18^F]nifene labeled brain regions of the thalamus, subiculum, cortex, striatum, hippocampus, and cerebellum were cut using the Leica CM1850 cryotome. The sections were air dried and exposed to phosphor films overnight. Films were read using the Cyclone Phosphor Imaging System. Region-of-interest (ROI) of same size were drawn and analyzed on brain regions rich in α4β2* nicotinic receptors using OptiQuant software and binding of [^18^F]nifene measured in Digital Light Units/mm^2^ (DLU/mm^2^).

#### 2.7.2. In Vitro Mice Brain Autoradiography

Mice were decapitated, the brain was rapidly removed and frozen in isopentane at −20 °C. Sagittal sections (10 μm thick) containing the cortex, striatum, thalamus, hippocampus and cerebellum were prepared using LEICA CM 1850 cryotome at −20 °C and stored at −80 °C until use. For binding studies, slides were thawed for approx. 15 min at ambient temperature and were subsequently pre-incubated for 10 min at ambient temperature in buffer (120 mmol/L Tris HCl containing 5 mmol/L NaCl, 5 mmol/L KCl, 2.5 mmol/L CaCl_2_, 1 mmol/L MgCl_2_, pH 7.4). The preincubation buffer was then discarded. Subsequently, the slices were treated with incubation buffer containing [^18^F]nifene (148 kBq/mL) at 37 °C for 60 min. Competitive binding assay with different concentrations of unlabeled nifene (0.01, 0.1, and 1 μM) were carried out. Nonspecific binding was measured in the presence of 300 μmol of nicotine. After incubation, slides were washed twice (2 min each) with ice-cold incubation buffer, followed by a quick rinse in cold (0–5 °C) deionized water. The dried slides were apposed to phosphor screens and read by the Cyclone Phosphor Imaging System (Packard Instruments Co.). The amount of bound [^18^F]nifene in the autoradiograms was evaluated in various brain regions (as digital lights units (DLU]/mm^2^) using the OptiQuant acquisition and analysis program (Packard Instruments Co.).

### 2.8. Hualpha-Syn ((A53T) Mice

Male (n = 4) and female (n = 4), hemizygous Hualpha-Syn ((A53T) and no-carrier male(n = 4) and female (n = 4) mice were used in the study. All mice were injected [^18^F]nifene (PETNET solutions) intraperitoneally in normal saline (7.4 ± 0.7 MBq in 0.05–0.1 mL sterile saline) under 3% isoflurane (Patterson Veterinary, Loveland, CO, USA). Mice were then awake after [^18^F]nifene injections and free to move in their cages for 2 h. They were placed in the supine position in a mouse holder and anesthetized with 3% isoflurane for whole-body PET/CT imaging. A 15 min-long PET scans was acquired 2 h after [^18^F]nifene injections followed by a 10-min-long CT scan after the PET scan for attenuation correction and anatomical delineation of PET images. The Inveon Multimodality scanner was used for all combined PET/CT experiments.

### 2.9. Image Analysis

All in vivo images were analyzed using Inveon Research Workplace (IRW) software (version 4.2) (Siemens Medical Solutions, Knoxville, TN, USA) and PMOD Software (version 3.0) (PMOD Technologies, Zurich, Switzerland). Whole-body PET/CT images were analyzed using the IRW software for [^18^F]nifene uptake and any other CT anomalies in the whole body images. For brain quantitative analysis, brain images were analyzed using PMOD, with PET images co-registered to a mouse brain MRI template [28]. The magnitude of [^18^F]nifene was expressed as standard uptake value (SUV) which was computed as the average [^18^F]nifene activity in each volume of interest, VOI (in kBq/mL) divided by the injected dose (in MBq) times the body weight of each animal (in Kg). The SUV values were then statistically analyzed using students t-test and Hualpha-Syn ((A53T) PD mice were compared with non-carrier mice.

### 2.10. In Vitro Postmortem Human Brain Autoradiography

Human brain frontal cortex tissue from the 6 PD and 6 cognitively normal (CN) subjects were preincubated in Tris buffer (described above) for 15 min. The slides contained 1 to 3 brain sections each were placed in separate glass chambers (six slides per chamber). The preincubation buffer was discarded and then to the chambers, [^18^F]nifene in Tris buffer pH 7.4 (60 mL; 37 kBq/mL), was added and the chambers were incubated at 25 °C for 1 h. Nonspecific binding was measured in separate chambers in the presence of 300 μM nicotine. The slices were then washed with cold buffer twice, 3 min each time, Tris buffer and cold water for rinse. The brain sections were air dried, exposed overnight on a phosphor film, and then placed on the Phosphor Autoradiographic Imaging System/ Cyclone Storage Phosphor System (Packard Instruments Co.). Regions of interest (ROIs) were drawn on the slices and the extent of binding of [^18^F]nifene was measured in DLU/mm^2^ using the OptiQuant acquisition and analysis program (Packard Instruments Co.).

### 2.11. Immunohistochemistry

Immunostaining of all brain sections were carried out by University of California-Irvine, Pathology services using Ventana BenchMark Ultra protocols. Neighboring slices were immunostained for Ubiquitin (Cell Marque catalog no. 318A-18, Rocklin, CA, USA) and α-synuclein (EMD Millipore Corporation, lot No. 2985418, Burlington, MA, USA). All IHC stained slides were scanned using the Ventana Roche instrumentation and analyzed using QuPath.

## 3. Results

### 3.1. Synthesis

The precursor, 2-(trimethylamino)-3-[2-((*S*)-*N*-tert-butoxycarbonyl-3-pyrrolinyl)-methoxy]pyridine triflate 8 (TMAT) was successfully prepared in a two-step procedure starting with *N*-BOC-nifene using standard procedures for related compounds. Substitution of fluorine in *N*-BOC-nifene by dimethylamine provided moderate yields of dimethylamino-*N*-BOC-nifene in >50% yield. Methylation of the dimethylamino group resulted in the product, TMAT in low yields (~30%). The purified salt was found to be stable for use in radiolabeling reactions. The HPLC elution profiles of the two precursors, TMAT (retention time 5.5 min) and *N*-BOC-nitronifene (9.5 min) were significantly different as seen in Figure 2A,B).

### 3.2. Radiosynthesis

Nucleophilic fluorine-18 displacement was efficiently carried out on both the precursors. The major difference between the two precursors is the reaction times of fluorine-18 exchange and separation of precursors from the product. In the case of *N*-BOC-nitronifene radiolabeling reaction was carried out for 30 min, while for the TMAT precursor, the radiolabeling reaction was terminated at 15 min. The radiochemical yields for the two reactions were similar. The major difference was in the HPLC purification. The retention time of [^18^F]*N*-BOC-nifene was found to be approx. 10 min which was very close to the *N*-BOC-nitronifene precursor (9.5 min), whereas the TMAT precursor eluted much earlier and was distinctly separated from the radiolabeled product.

### 3.3. PET/CT Imaging

**Figure 3:**
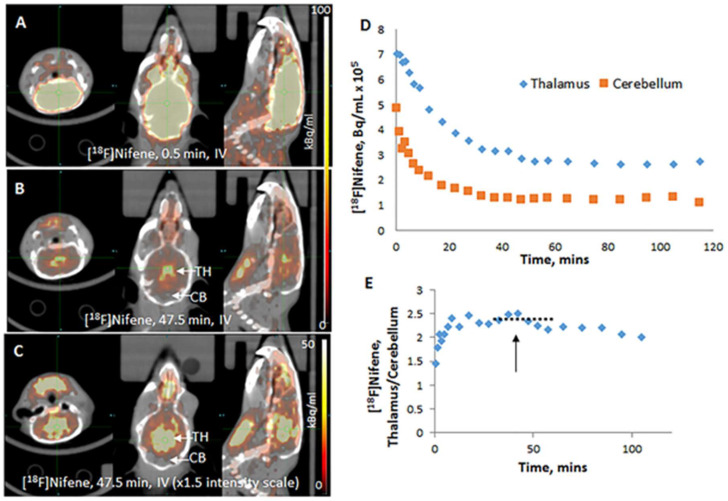
PET/CT of intravenous (IV) [^18^F]nifene (5.55 MBq in 20 mL saline) administered in wild-type mouse (BALB/c, female 24 g). (**A**) Coronal, transaxial, and sagittal images of mouse head at 0.5 min after injection showing high levels of [^18^F]nifene in the brain; (**B**) coronal, transaxial, and sagittal images of same mouse head at 47.5 min after injection; (**C**) PET intensity increased (×1.5) of images in (**B**) showing [^18^F]nifene bound the thalamus and cortical regions; (**D**) time–activity curve of thalamus and cerebellum of region-of-interest shown on the sagittal slices; (**E**) ratio of thalamus to cerebellum of time–activity curve showing plateau between 30 to 60 min (dotted line).

Intravenous (IV) [^18^F]Nifene: Rapid uptake of [^18^F]nifene was observed in all regions of the BALB/c mouse brain with levels of exceeding 1% injected dose/mL at 0.5 min iv post-injection (Figure 3A). Thalamic regions exhibited the highest retention as it has a maximum amount of α4β2* receptors (Figure 3B). Clearance of [^18^F]nifene from all brain regions were rapid. Intensified images show significant levels of uptake observed in the cortical regions and little binding was present in the cerebellum (Figure 3C). Time–activity curves of the thalamus and cerebellum in Figure 3D show initial rapid uptake in various brain regions followed by greater retention in the thalamus compared to cerebellum. Ratio of uptake for the thalamus versus reference region cerebellum reached a plateau at approx. 30 min post-injection. Maximal thalamus to cerebellum ratio was approx. 2.3 between 30 and 60 min (Figure 3E). This ratio gradually decreased over time to 2 by 120 min.

**Figure 4:**
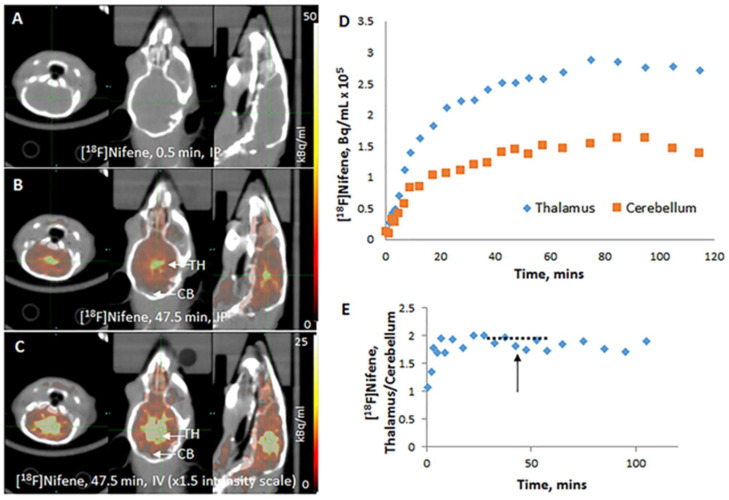
PET/CT of intraperitoneal (IP) [^18^F]nifene (5.77 MBq in 50 mL saline) administered in wild-type mouse (BALB/c, female 24 g). (**A**) Coronal, transaxial, and sagittal images of mouse head at 0.5 min after injection showing no [^18^F]nifene in the brain; (**B**) coronal, transaxial, and sagittal images of same mouse head at 47.5 min after injection; (**C**) PET intensity increased (×1.5) of images in (**B**) showing [^18^F]nifene bound the thalamus and cortical regions; (**D**) time–activity curve of thalamus and cerebellum of region-of-interest shown on the sagittal slices; (**E**) ratio of thalamus to cerebellum of time–activity curve showing plateau between 30 to 60 min (dotted line).

Intraperitoneal (IP) [^18^F]Nifene: Uptake of [^18^F]nifene was gradual and very little brain activity was observed at 0.5 min post IP injection in the BALB/c mouse (Figure 4A). Uptake reached high levels (approx. 0.5% injected dose/mL) after 30–40 min post-injection in the thalamus as well as the cerebellum. Thalamus was clearly visualized and exhibited the highest retention of [^18^F]nifene (Figure 4B). Clearance of [^18^F]nifene from brain regions occurred after 90 min post-injection. Significant levels of uptake was observed in the cortical regions and little binding was present in the cerebellum (Figure 4C). Time–activity curves in Figure 4D show slow uptake in various brain regions followed by greater retention in the thalamus compared to cerebellum. Ratio for the thalamus versus reference region cerebellum reached a plateau at approx. 30 min post-injection. Maximal thalamus to cerebellum ratio was approx. 2 between 30 and 60 min and remained at that level until the end of the scan at 120 min (Figure 4E).

### 3.4. [^18^F]Nifene Imaging: In Vivo, Ex Vivo, and In Vitro

**Figure 5:**
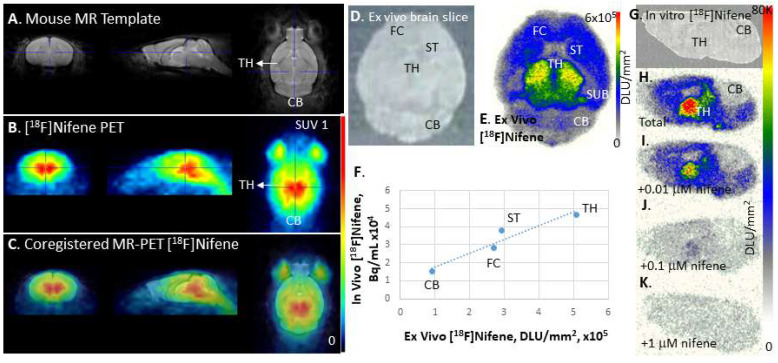
PET-MR of [^18^F]nifene: (**A**) Mouse brain MR template showing coronal, sagittal and transaxial slices, with cross hairs placed on the thalamus; (**B**) [^18^F]nifene PET coronal, sagittal, and transaxial slices, 30 min post IP injection (5.50 MBq in 100 mL saline) in wild-type mouse (C57BL/6, male 28 g); (**C**) [^18^F]nifene PET co-registered with mouse MR template (**A**,**B**); (**D**) mouse brain slice ex vivo 30 μm thick; (**E**) ex vivo autoradiograph of [^18^F]nifene of mouse slice (**D**), after PET experiment in (**B**), showing regions-of-interest for analysis; (**F**) comparison of [^18^F]nifene in vivo PET and [^18^F]nifene ex vivo brain slice autoradiograph (**B** vs. **E**) using four brain regions; (**G**–**K**) in vitro [^18^F]nifene binding in 10 mm sagittal mice brain (**G**) slices showing [^18^F]nifene binding (**H**; 100%) and effect of 0.01 mM (**I**; 72%), 0.1 mM (**J**; 15%), and 1 mM (**K**; 6%) unlabeled Nifene.

MRI Co-Registration of [^18^F]nifene PET: For detailed analysis of mouse brain [^18^F]nifene binding and translation to mouse model studies, C57BL/6 mice were injected intraperitoneally. Similar to outlined in Section 3.3, the acquired PET/CT image showed uptake of [^18^F]nifene in the brain. The summed mouse [^18^F]nifene PET image of the brain (Figure 5B) was co-registered with the mouse brain template (Figure 5A) using PMOD [29]. High binding region of [^18^F]nifene corresponded to thalamus in the MRI. Lower levels of binding were seen in the striatum and cortex. The lowest levels appeared to be in the cerebellum. The bilateral anterior regions corresponded to the retinal portions of the eye.

Ex vivo [^18^F]nifene mouse brain analysis: For evaluating ex vivo binding, after the PET scan, the C57BL/6 mouse was killed and the brain was excised and sectioned. A 20 mM horizontal brain section is shown in Figure 5D along with [^18^F]nifene binding in this slice shown in Figure 5E. Binding of [^18^F]nifene in the ex vivo brain slice followed the general pattern on in vivo binding. Thalamus was clearly the high binding region. Striatum and cortex exhibited moderate levels of binding consistent with in vivo findings. Smaller regions such the subiculum were seen in the ex vivo image but were not clearly visualized in the in vivo scans. Cerebellum had the least amount of activity both in vivo and ex vivo. [^18^F]Nifene binding correlated well between in vivo and ex vivo methods of imaging in select brain regions, as seen in Figure 5F.

In vitro [^18^F]nifene mouse brain competition: Binding of [^18^F]nifene in mouse brain (C57BL/6) sagittal brain sections (Figure 5H) followed a similar trend as seen in the ex vivo brain slice seen in Figure 5E. Thalamus had the highest levels, while cerebellum had the least amount of [^18^F]nifene binding. Significant levels of [^18^F]nifene binding was observed in regions of the subiculum, striata and cortex. Binding in the hippocampus were low. This in vitro distribution of [^18^F]nifene was similar to the ex vivo distribution described above. This binding profile is similar to in vitro [^18^F]nifene rat brain distribution [4]. Unlabeled nifene progressively reduced the binding of [^18^F]nifene and at 1 mM nifene (Figure 5K), all [^18^F]nifene was reduced to nonspecific binding levels. At 10 nM nifene concentrations (Figure 5I), 30% of receptors were occupied and the occupancy increased to 85% at 100 nM nifene (Figure 5J).

### 3.5. A53T PD Mice Model—In Vitro [^18^F]Nifene Studies

**Figure 6:**
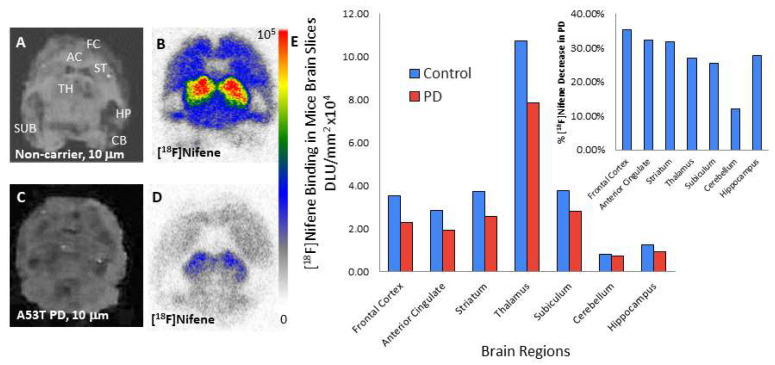
In vitro [^18^F]nifene in Hualpha-Syn ((A53T) PD mice model: (**A**) Non-carrier mouse brain slice 10 μm thick; (**B**) in vitro autoradiograph of [^18^F]nifene of non-carrier mouse brain slice; (**C**) A53T PD mouse brain slice; 10 μm thick; (**D**) in vitro autoradiograph of [^18^F]nifene of Hualpha-Syn ((A53T) mouse brain slice; (**E**) comparison of [^18^F]nifene in non-carrier mice (n = 3) and Hualpha-Syn ((A53T) PD mice (n = 3) in different brain regions. Inset in (**E**) shows percent decrease of [^18^F]nifene binding in A53T PD mice compared to non-carrier mice (*p* < 0.05 for frontal cortex, anterior cingulate, striatum, and thalamus; not significant for subiculum, cerebellum, and hippocampus).

Brain sections from non-carrier mice (Figure 6A) and Hualpha-Syn ((A53T) PD mice (Figure 6C) were treated with [^18^F]nifene to assess effects of α-synuclein aggregates. Presence of α-synuclein aggregates were confirmed by anti-α-synuclein immunostaining [20]. Binding of [^18^F]nifene in the non-carrier mice brain sections (Figure 6B) followed a similar trend of thalamus having the highest binding and cerebellum the least, similar to that described in Figure 5. Frontal cortex, anterior cingulate, striatum and subiculum had moderate levels of binding. Levels in hippocampus were slightly higher than in the cerebellum. In the A53T PD mice, [^18^F]nifene binding was reduced in most regions (Figure 6D). Except the cerebellum, reductions in [^18^F]nifene binding were over 25% (Figure 6E inset). Frontal cortex, anterior cingulate, and striatum showed the greatest reductions (>30%).

### 3.6. A53T PD Mice Model-In Vivo PET/CT [^18^F]Nifene Studies

**Figure 7:**
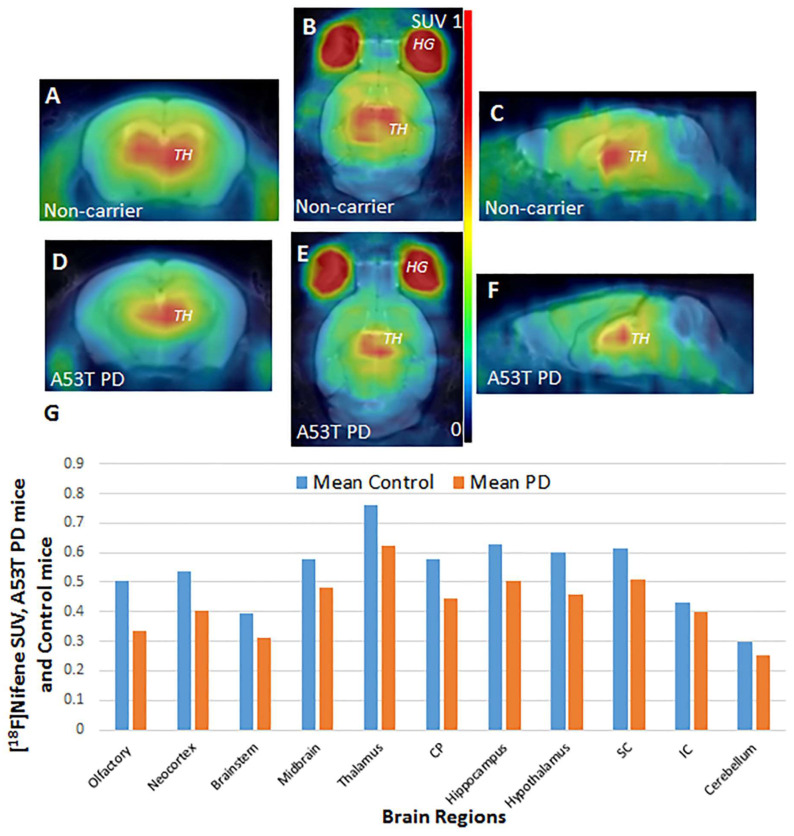
Hualpha-Syn ((A53T) PD mice brain PET-MR of [^18^F]nifene. (**A**–**C**): [^18^F]Nifene PET co-registered to mouse MR template non-carrier mice; (**D**–**F**): [^18^F]Nifene PET co-registered to mouse MR template Hualpha-Syn ((A53T) PD mice; (**G**): Plot of [^18^F]nifene SUV of Hualpha-Syn ((A53T) PD mice versus non-carrier mice in different brain regions (n = 4 WT and n = 4 TG). CP = caudate putamen; SC = superior colliculus; IC = inferior colliculus (*p* < 0.05 for all regions except brain stem, midbrain, and hippocampus, SC, IC, and cerebellum were not significant).

Non-carrier mice and Hualpha-Syn ((A53T) PD mice were injected [^18^F]nifene intraperitoneally. Similar to outlined in Section 3.3, the acquired PET/CT image showed uptake of [^18^F]nifene in the brain. The summed mouse [^18^F]nifene PET images of the brain of the non-carrier mice (Figure 7A–C) and the A53T PD mouse (Figure 7D–F) were co-registered with the mouse brain template using PMOD. The high uptake in the bilateral regions outside of the brain corresponded to the regions of the eyes and harderian glands. High uptake of other PET radiotracers have been previously observed in these regions. High binding region of [^18^F]nifene in the brain corresponded to thalamus in the MRI.

The overall regional brain distribution of [^18^F]nifene in the non-carrier and A53T PD mice was similar, except that the amounts of bound [^18^F]nifene in the A53T PD mice was lower. Standard uptake values (SUV) of [^18^F]nifene were computed for the various brain regions in the non-carrier mice and the A53T PD mice using our previously reported mouse brain template [23]. Ratio of SUV of A53T PD mice over the non-carrier mice for various brain regions are shown in Figure 7G. Several brain regions exhibited greater than 20% reductions in [^18^F]nifene binding in the A53T PD mice brains. These included olfactory bulb, neocortex, brainstem, caudate putamen (striatum) and hippocampus. Reductions in the thalamus, midbrain, colliculi (superior and inferior), and cerebellum were lower.

### 3.7. In Vitro Postmortem PD Human Brain [^18^F]Nifene Autoradiography

**Figure 8:**
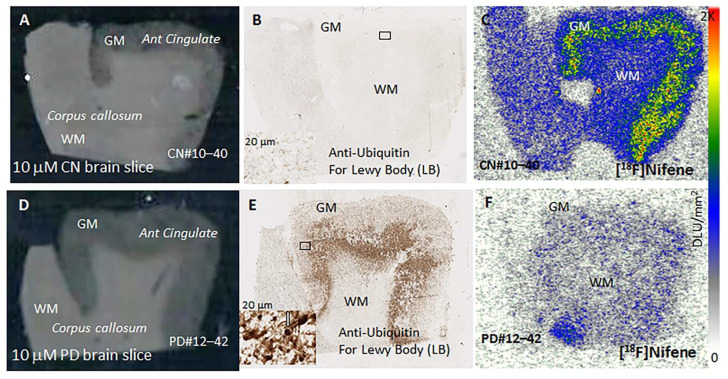
Comparison of [^18^F]nifene in post-mortem human PD versus controls. (**A**) CN brain section, 10 micron thick consisting of grey matter (GM) anterior cingulate (AC) and white matter (WM) corpus callosum (CC); (**B**) ubiquitin IHC for LB; (**C**) [^18^F]nifene binding in adjacent section; (**D**) PD brain section, 10 micron thick consisting of grey matter (GM) anterior cingulate (AC) and white matter (WM) corpus callosum (CC); (**E**) ubiquitin IHC for LB; inset at 20 mm shows LB and measured diameter of LB in inset was 6 to 9 microns; (**F**) [^18^F]nifene binding in adjacent section showing reduced binding compared to CN brain.

**Figure 9:**
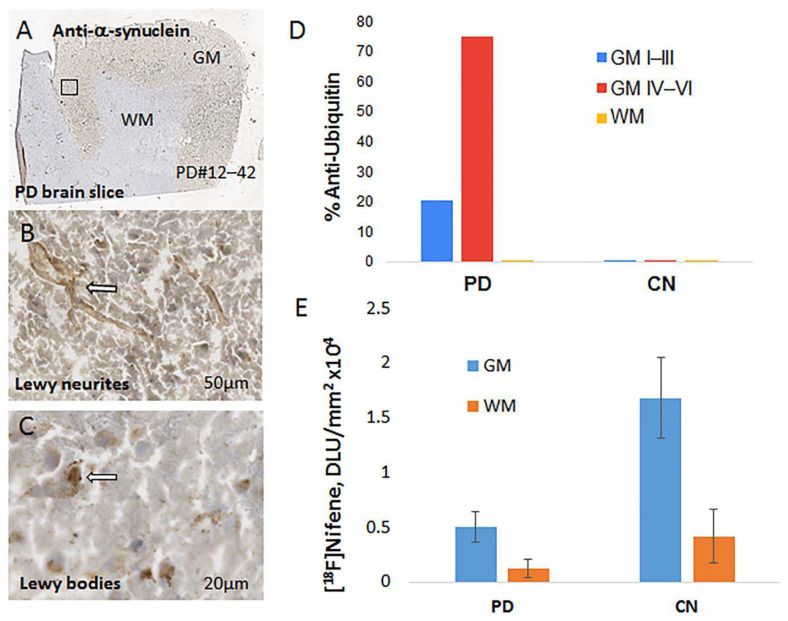
(**A**) Anti-α-synuclein IHC of PD brain slice; (**B**) magnified view at 50 μm showing presence of Lewy neurites; (**C**) Lewy bodies stained for α-synuclein in Lewy bodies see at 20 μm; (**D**) anti-ubiquitin staining showing presence of Lewy bodies in the PD and absent in the CN brains (*p* < 0.001). Cortical layers IV–VI had significantly greater amounts compared to outer I–III layers. White matter did not reveal presence of Lewy bodies; (**E**) comparison of [^18^F]nifene in post-mortem human PD versus controls showing decreases in PD GM (*p* < 0.01).

Postmortem human brain sections from control subjects (Figure 8A) and PD subjects (Figure 8D) consisting of anterior cingulate and corpus callosum from six subjects in each group were used for [^18^F]nifene autoradiography. Brain slices form all subjects in the two groups were immunostained with anti-ubiquitin for Lewy bodies. In the control subjects (Figure 8B) there was no staining with anti-ubiquitin, thus confirming the absence of Lewy bodies. In the case of the PD subjects, there was significant staining by anti-ubiquitin, confirming the presence of Lewy bodies (Figure 8E). The inner cortical layers (IV to VI) had greater levels of staining compared to outer layers (I to III) (Figure 8E and Figure 9D). Inset in Figure 8E shows a close of view of the Lewy bodies whose diameter was measured to be 6 to 9 microns (magnification at 20 μm). White matter regions in both control and PD subjects did not exhibit any significant amount of anti-ubiquitin staining (Figure 8B,E). Binding of [^18^F]nifene in the anterior cingulate grey matter region was observed in the control brains, with binding greater in the inner cortical layers (Figure 8C). Compared to control brains, PD brains exhibited significantly lower [^18^F]nifene binding (Figure 8F), both in grey matter and white matter.

Adjacent brain sections were further labeled with anti-α-synuclein in order to confirm α-synuclein aggregates (Figure 9A). Closer views of the brain section at 50 μm confirmed presence of Lewy neurites (Figure 9B) and at 20 μm Lewy bodies with α-synuclein aggregates were identified (Figure 9C). Aggregates of α-synuclein were uniformly spread out through the various cortical layers (Figure 9A), while the anti-ubiquitin staining for Lewy bodies occurred more in the inner cortical layers (Figure 8E). Figure 9D shows levels of Lewy bodies in the grey matter and white matter of PD and CN brains. Very little anti-ubiquitin was observed in the CN brains, but in PD brains the inner cortical layers exhibited the highest levels (>70%) and lower levels were observed in outer layers (~20%). Overall, [^18^F]nifene binding in all the PD subjects was lower compared to control subjects (Figure 9E). Ratio of [^18^F]nifene in CN GM versus PD GM was found to be >3, suggesting a significant decrease in [^18^F]nifene binding in PD. [^18^F]nifene binding was reduced in the white matter of PD subjects as well, compared to CN subjects, but not as significantly as in the GM.

## 4. Discussion

Among the various PET imaging agents for α4β2* nAChRs currently being used in humans, [^18^F]nifene exhibits the fastest equilibration in vivo [5]. Our previous studies in rats [10], monkeys [4,11] and humans [8] have validated interspecies similarity of in vivo behavior of [^18^F]nifene. Previous whole body radiation dosimetry of [^18^F]nifene in mice did not include mice brain studies in detail [29]. With the development of several transgenic mice models of neurodegenerative disorders, availability of [^18^F]nifene for evaluating changes in α4β2* nAChRs offers a unique tool. The goal in this work was, therefore, to develop methodology for [^18^F]nifene studies in mice models and validate the utility of [^18^F]nifene in PD.

Previous methods of radiosynthesis of [^18^F]nifene involved the use of fluorine displacement of a nitro group in the precursor (Figure 2). Purification by chromatographic separation of the nitro precursor from the fluorinated product was challenging due to their very close retention times (Figure 2B,C). In order to improve the separation, the alternate trimethylammonium precursor was prepared (Figure 2). The precursor was synthesized in two steps from *N*-BOC-nifene, by displacement of the fluorine with dimethylamine followed by methylation of the tertiary amine using methyl trifluorosulfonic acid. Retention time of this trimethylammonium precursor was approximately 5 min (Figure 2A), thus eluting much before the desired *N*-BOC-[^18^F]nifene product and enabling good purification. Radiochemical yields of the two precursors (nitro and trimethylamine) were comparable under reaction similar conditions of temperature and reaction times. The distinct advantage of the new precursor was the purification of the intermediate.

After intravenous administration of [^18^F]nifene in the mouse, brain uptake was rapid (Figure 3A). The unbound radiotracer cleared quickly from the thalamus and other receptor poor regions such as the cerebellum. Thalamus exhibited greater retention (Figure 3B) and other extrathalamic regions were also seen to bind the radiotracer (Figure 3C). Time–activity curves in the thalamus and cerebellum showed the rapid kinetics of [^18^F]nifene and the greater retention in the thalamus (Figure 3D). A ratio plot of thalamus to cerebellum showed a maximum of approximately 2.5 (Figure 3E). A plateau of the ratio was observed between 30 and 60 min, after which there was a gradual decrease in the ratio. Distinct difference after intraperitoneal administration of [^18^F]nifene in the mouse were observed compared to intravenous administration (Figure 4). Initial images show no uptake of [^18^F]nifene in the brain (Figure 4A). The radiotracer is taken up gradually over time with greater binding observed in the thalamus lower levels in the cerebellum (Figure 4B). Thalamus exhibited greater retention (Figure 4B) and other extrathalamic regions were also seen to bind the radiotracer (Figure 4C). Time–activity curves in the thalamus and cerebellum showed the slow uptake of [^18^F]nifene and the greater retention in the thalamus (Figure 4D). A ratio plot of thalamus to cerebellum showed a maximum of approximately 2 (Figure 4E). A plateau of the ratio was observed between 30 and 60 min, after which there was a gradual decrease in the ratio.

Because of the need to carry out longitudinal PET studies in transgenic mice and the difficulty in repeat intravenous administrations, the intraperitoneal administration of [^18^F]nifene in mice is preferred. Our findings suggest a similarity in the time course of [^18^F]nifene in the brain where a static scan 30 to 60 min post intraperitoneal administration would be suitable. The thalamus to cerebellum ratio in the case of intraperitoneal administration was found to be about 20% lower compared to the intravenous administration.

Further detailed analysis of intraperitoneally administered [^18^F]nifene bound to the mouse brain was carried out using co-registration of PET with mouse brain MR template (Figure 5A–C). The in vivo measures were correlated with ex vivo brain slices of the same mouse (Figure 5D,E). Thalamic and extrathalamic regions were confirmed using the PET-MR co-registered images, including the extracranial localization in the vicinity of the eyes (Figure 5C). Ex vivo brain autoradiography showed more detailed localization of [^18^F]nifene in various brain regions (Figure 5). Thalamus had the highest levels followed by subiculum, striatum, frontal cortex and the lowest in the cerebellum. In vivo measures of thalamus, striatum, frontal cortex and cerebellum correlated well with the ex vivo measures suggesting reliability of in vivo measures for comparative, longitudinal studies in transgenic mice models. In vitro [^18^F]nifene binding in mouse brain slices (Figure 5G,H) correlated with ex vivo regional distribution. Unlabeled nifene progressively displaced [^18^F]nifene and at 1 mM was able to completely displace from all brain regions confirming reversibility (Figure 5I–K). Our previous studies in rats confirmed absence of any radiolabeled metabolites in the brain after [^18^F]nifene administration [10].

The Hualpha-Syn ((A53T) transgenic line of mice are of utility when studying Parkinson’s disease and various synucleinopathies. The transgenic mice used in the study express a A53T missense mutant form of human α-synuclein under the control of the murine prion promoter. The transgene yielded a 48,317 bp deletion in 2310039L15Rik by integrating into chromosome 10. The Hualpha-Syn ((A53T) mice exhibit the familial Parkinson’s disease- associated A53T missense mutant form of human α-synuclein (α-Syn), and express the A53T mutant α-Syn at a level sixfold that of the endogenous mice α-Syn. The age at which the hemizygous mice spontaneously develop neurodegenerative disease is between nine to 16 months of age. Neuronal abnormalities displayed by affected mice include pathological accumulations of α-Syn and ubiquitin. α-Syn-dependent neurodegeneration associated with increased/abnormal detergent-insoluble α-Syn and α-Syn aggregation is shown in brain regions as well. In the A35T α-Syn mutant mice, it was discovered that they attain intraneuronal inclusions, mitochondrial degeneration, and cell death in neocortex, brainstem, and spinal cord. In addition, they formed inclusions similar to Lewy bodies in neurons, and had profound deficits in their motor neurons, which could explain their paralysis [22].

[^18^F]Nifene binding in noncarrier mice brains followed the normal distribution as described above in C57BL\6 mice (Figure 6A,B). Thalamus was the highest followed by subiculum, striatum and frontal cortex. Cerebellum was the lowest as expected. Two additional regions, namely anterior cingulate and hippocampus, were also analyzed. Binding in the hippocampus was low, while anterior cingulate was closer to levels of the frontal cortex. In the Hualpha-Syn ((A53T) PD mice brains, there was overall reduction of the binding of [^18^F]nifene in most brain regions (Figure 6D, E). Frontal cortex, anterior cingulate and striatum showed a decrease of more than 30%, while thalamus, subiculum and hippocampus were over 25% (Figure 6E inset). The reduction in [^18^F]nifene binding may be related to the α-synuclein aggregates in Hualpha-Syn ((A53T) PD brain slices. In [^18^F]nifene PET/CT studies of non-carrier (Figure 7A–C) and Hualpha-Syn ((A53T) PD mice (Figure 7D–F), a similar reduction of [^18^F]nifene binding was observed in various brain regions. A reduction of SUV of Hualpha-Syn ((A53T) PD compared to non-carrier mice (Figure 7G) with some regions such as the cortex, caudate putamen and thalamus showing >20% decrease. The Hualpha-Syn ((A53T) PD mice have been shown to accumulate α-synuclein aggregates in these brain regions and spinal cord, potentially affecting other neurotransmitter receptor systems [30], resulting in motoric and nonmotoric deficits. Our previous [^18^F]FDG PET/CT studies in these Hualpha-Syn ((A53T) PD mice also observed significant metabolic deficits in these brain regions and spinal cord, contributing to hind limb muscle hypometabolism and leading to hind limb paralysis [23]. 

Postmortem human brain sections of the anterior cingulate from well characterized subjects were confirmed as controls (Figure 8A,B) and PD (Figure 8D,E) by the absence or presence of Lewy bodies. Anti-ubiquitin stained Lewy bodies were found in the inner cortical layers (Figure 8E) with dimensions of approximately 6–9 microns (Figure 8E inset). In the control brains, there was no anti-ubiquitin staining confirming the absence of Lewy bodies (Figure 8B and Figure 9D). Binding of [^18^F]nifene was observed in the grey matter regions within anterior cingulate (Figure 8C). In the case of the PD brain, this anterior cingulate binding of [^18^F]nifene was remarkably reduced. Even white matter binding of [^18^F]nifene in the PD brains was reduced compared to the control brains. The presence of α-synuclein aggregates in the anterior cingulate of the PD brain slice is shown in Figure 9A confirming the presence of Lewy neurites and Lewy bodies (Figure 9B,C). Reduction of [^18^F]nifene in the PD brain was significant compared to the control brains as seen in Figure 9E. PET studies in PD subjects using [^18^F]2-FA85380 have shown reduced levels of α4β2* nAChRs [31]. Human PET studies using [^18^F]nifene in PD subjects may be a valuable tool to study changes in the α4β2* nAChRs.

## 5. Conclusions

[^18^F]Nifene binding to α4β2* nAChRs is reduced in Hualpha-Syn ((A53) PD transgenic mice as well as human postmortem PD brains containing anterior cingulate compared to normal controls. Thus, [^18^F]nifene offers a valuable tool for PET imaging studies of PD.

## Data Availability

The data that support the findings of this study are available from the corresponding author upon reasonable request.
